# Endoscopic Complete Response to Zolbetuximab for Advanced Gastric Cancer With Claudin 18.2 Positive: A Case Report

**DOI:** 10.1002/deo2.70189

**Published:** 2025-08-22

**Authors:** Akinori Sasaki, Tomohiro Yamaba, Ayu Tachibana, Rika Kimura, Gaku Uchiyama, Tsubasa Yoshioka, Risa Okamoto

**Affiliations:** ^1^ Department of Gastroenterology Tokyo Bay Urayasu Ichikawa Medical Center Chiba Japan; ^2^ Department of Oncology Tokyo Bay Urayasu Ichikawa Medical Center Chiba Japan

**Keywords:** cldn18.2 | endoscopic complete response | gastric cancer | immune checkpoint inhibitor | zolbetuximab

## Abstract

Recently, zolbetuximab combined with chemotherapy has been approved as a first‐line treatment for advanced gastric cancer (GC). However, to date, no endoscopic images demonstrating endoscopic complete response (eCR) to zolbetuximab plus chemotherapy have been reported. Herein, we report the case of an 80‐year‐old man who presented with epigastric pain and loss of appetite and was diagnosed with undifferentiated GC and peritoneal metastasis. The patient received modified FOLFOX6 plus zolbetuximab because genetic testing revealed HER2‐negative and claudin 18.2‐positive. He experienced grade 2 nausea, which improved with the administration of antiemetic agents. Computed tomography performed at month 5 showed resolution of peritoneal dissemination. Endoscopic examination during the same period showed the disappearance of the primary gastric tumor with scarring. Biopsy of the scar area revealed only inflammatory cells; therefore, the primary lesion was defined as an eCR. To the best of our knowledge, this is the first report of eCR to zolbetuximab and chemotherapy in a patient with GC and claudin 18.2 positive. This case report suggested that chemotherapy combined with zolbetuximab may offer greater therapeutic benefit than chemotherapy combined with immune checkpoint inhibitors in patients with undifferentiated GC. Further analysis is expected to enable the identification of patients with claudin 18.2‐positive GC in whom zolbetuximab treatment is effective.

## 1 Introduction

Gastric cancer (GC) is the third leading cause of cancer‐related mortality worldwide [1]. Several chemotherapeutic regimens, including platinum, fluoropyrimidine, taxanes, and irinotecan, have been approved for the treatment of advanced GC [2]. Additionally, molecular targeted drugs (e.g., trastuzumab and ramucirumab) and immune checkpoint inhibitors (e.g., nivolumab and pembrolizumab) have been proven to have antitumor effects and have been approved for patients with GC.

Recently, zolbetuximab, a monoclonal antibody targeting claudin‐18 isoform 2 (CLDN18.2), showed improved benefits when combined with standard chemotherapy in patients with CLDN18.2‐positive GC in two randomized phase 3 trials [3, 4]. In these two trials, the objective response rate to chemotherapy plus zolbetuximab was 50–‐60%. However, only a few cases have shown complete response, and there have been no reports of endoscopic complete response (eCR).

Herein, we report a case of CLDN18.2‐positive GC in which eCR to zolbetuximab was observed. The patient provided informed consent for the presentation of anonymized clinical information.

## 2 Case Report

**FIGURE 1:**
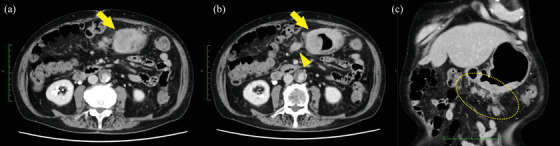
Abdominal computed tomography (CT) and endoscopic findings at diagnosis. Abdominal CT (a–c) shows an unevenly strengthened wall of the lower body of the stomach (arrow), lymph node metastases (arrowhead), and peritoneal dissemination around the stomach (dotted circle).

**FIGURE 2:**
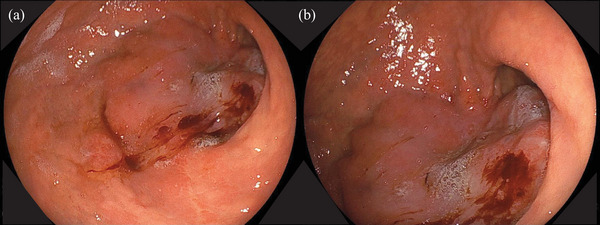
Upper gastrointestinal endoscopy (a, b) reveals Borrmann type 3 gastric carcinoma in the greater curvature of the antrum of the stomach.

**FIGURE 3:**
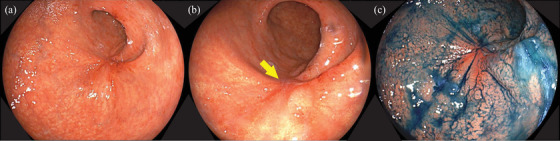
Upper endoscopy shows that the primary lesion has disappeared and can be observed as a relatively gentle mucosa (arrow). A biopsy specimen obtained from the scarred primary lesion reveals the presence of only inflammatory cells, and no tumor cells are found.

**FIGURE 4:**
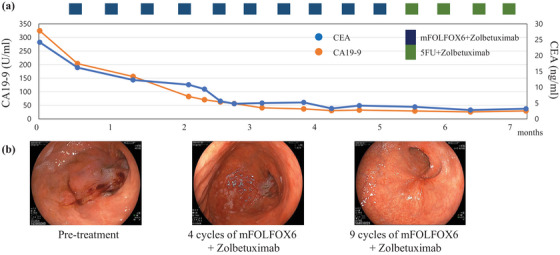
Course of tumor markers (carcinoembryonic antigen [CEA] and carbohydrate antigen 19‐9 [CA 19‐9]) while receiving treatment with mFOLFOX6 and Zolbetuximab (a). Upper endoscopy showing Borrmann type 3 gastric carcinoma pre‐mFOLFOX6 Plus Zolbetuximab, decreased tumor volume after 4 cycles of mFOLFOX6 plus Zolbetuximab, and tumor disappearance after 9 cycles of mFOLFOX6 plus Zolbetuximab (b, c).

An 80‐year‐old man with epigastric pain and loss of appetite was diagnosed with advanced GC with peritoneal dissemination in October 2024. The patient had a medical history of hypertension. Laboratory investigations revealed iron‐deficiency anemia and elevated levels of carcinoembryonic antigen (24.2 ng/mL) and carbohydrate antigen 19‐9 (324.6 ng/mL). Abdominal computed tomography (CT) revealed an unevenly strengthened wall in the lower body of the stomach, lymph node metastasis, and peritoneal dissemination around the stomach (Figure 1). Upper gastrointestinal endoscopy revealed Borrmann type 3 gastric carcinoma in the anterior stomach (Figure 2). Biopsy of the primary tumor revealed moderately and poorly differentiated adenocarcinoma. The patient was diagnosed with stage IV gastric carcinoma (clinical stage T3N1M1, according to the TNM classification, eighth edition). Subsequently, therapy‐related target genes were detected: HER2 staining was negative; *MLH1*, *MSH2*, *MSH6*, and *PMS2* were positive; Combined Positive Score of Programmed Death‐Ligand 1 (clone number 28‐8) staining was ≥ 5%; and CLDN18.2 was positive (defined as ≥75% of tumor cells showing moderate‐to‐strong membranous CLDN18 staining, determined by using the investigational VENTANA CLDN18 RxDx Assay). He received first‐line chemotherapy with a modified FOLFOX6 plus zolbetuximab regimen every 2 weeks: oxaliplatin, 85 mg/m^2^; leucovorin, 400 mg/m^2^; 5‐fluorouracil, bolus 400 mg/m^2^ and continuous infusion at 2400 mg/m^2^ over 46 h; and zolbetuximab, 800 mg/m^2^ (400 mg/m^2^ for the second and subsequent cycles). The patient had grade 1 nausea and dysgeusia owing to the regimen containing zolbetuximab, but tolerated the dose. However, the patient's general condition gradually improved, with an associated reduction in the severity of epigastric pain. CT on day 56 revealed significant shrinkage of the primary lesion and disappearance of peritoneal dissemination, corresponding to a non‐complete response/non‐progressive disease according to the Response Evaluation Criteria in Solid Tumors version 1.1. Endoscopic examination was performed during the same period, and the primary tumor in the stomach was in a state of shrinkage, with no findings of active bleeding or intestinal stenosis (Figure S1). Based on these findings, chemotherapy, including zolbetuximab, was continued. The second evaluation of the therapeutic effects using CT 5 months after treatment initiation showed no findings indicating an increase in primary lesions or metastases. Furthermore, endoscopic examination during the same period showed that the primary lesion had disappeared and could be observed as relatively gentle mucosa (Figure 3). A biopsy specimen was obtained from the scarred primary lesion; however, only inflammatory cells were detected, and no tumor cells were found. Based on these findings, the primary lesion was defined as eCR, according to the Japanese Classification of Gastric Carcinoma, 15th Edition [5]. The oxaliplatin was discontinued from the 11th course onwards due to worsening of his peripheral neuropathy. As of April 2025, the patient has been receiving 5‐fluorouracil plus zolbetuximab for more than 7 months, with no evidence of tumor progression or elevation in tumor markers (Figure 4).

## 3 Discussion

Here, we report the case of a patient with CLDN18.2‐positive GC treated with zolbetuximab. To the best of our knowledge, this is the first report of eCR to zolbetuximab in a patient with CLDN18.2‐positive GC.

CLDN18.2 protein, namely, a member of the tight junction protein family, is a highly selective biomarker with limited expression in normal tissues and often exhibits abnormal expression during the onset and development of several primary malignancies, including gastric, colorectal, breast, liver, bronchial, and non‐small cell lung cancers [6]. Zolbetuximab, a monoclonal antibody against CLDN18.2, exhibits antitumor effects through antibody‐dependent cell‐mediated cytotoxicity and complement‐dependent cell‐mediated cytotoxicity [6]. Two phase 3 trials, the SPOTLIGHT and GLOW trials, were conducted to evaluate the efficacy of zolbetuximab in patients with CLDN18.2‐positive GC [3, 4]. The results of these trials showed that zolbetuximab combined with first‐line chemotherapy resulted in improved progression‐free survival and overall survival benefits compared with chemotherapy alone.

Routine endoscopic examinations are not essential for assessing cancer progression in patients with advanced GC during chemotherapy. However, endoscopic procedures may be performed if biopsy of the tumor tissue is required or if there are symptoms related to GC (e.g., bleeding and intestinal stricture). In our case, endoscopic examination was performed to evaluate the primary lesion because no malignant disease was found on CT. The efficacy of chemotherapy was determined based on the Japanese Classification of Gastric Carcinoma, 15th Edition [5]. eCR was defined as the disappearance of all tumors and the absence of cancer cells detected on biopsy. We considered our case to be an eCR because it met these criteria. The efficacy of chemotherapy is usually assessed using CT or magnetic resonance imaging to evaluate the progression of not only the primary lesion but also any metastases. However, in patients with GC, symptoms (e.g., anemia and epigastric pain) are expected to improve as the primary tumor shrinks or disappears.

Currently, zolbetuximab or immune checkpoint inhibitors can be used in combination with cytotoxic chemotherapy in patients with HER2‐negative and CLDN18.2‐positive GC [3, 4, 7, 8]. Currently, the combination of zolbetuximab and an immune checkpoint inhibitor that is effective in patients with GC remains unknown. Therefore, attending physicians should select the most appropriate treatment regimen for these patients. The CheckMate 649 trial, which demonstrated the efficacy of nivolumab combined with chemotherapy, reported that treatment efficacy in the diffuse type was inferior to that in the intestinal type, based on histology in a subgroup analysis [7]. In contrast, studies on zolbetuximab plus chemotherapy showed that therapeutic effects in the diffuse type were equivalent to those in the intestinal type [3, 4]. Based on these results, we selected chemotherapy combined with zolbetuximab for our patient. Zolbetuximab is known to have a high incidence of adverse effects, such as nausea and vomiting. Its tolerability in older patients is of concern. However, the rate of early discontinuation due to adverse events such as nausea and vomiting was lower among Asian patients, especially in the Japanese subgroup [9]. These results suggest that the appropriate administration of antiemetics and good adherence are associated with low rates of treatment discontinuation. A combination of steroids, 5‐HT3 receptor antagonists, NK1 receptor antagonists, and olanzapine was administered at the initiation of chemotherapy to prevent nausea in our case. The implementation of these comprehensive antiemetic measures was considered to have contributed to treatment adherence and favorable clinical outcomes. A clinical trial combining zolbetuximab and nivolumab with chemotherapy is currently underway, and this regimen may become the standard treatment for patients with advanced GC in the future [10].

Although the present report details only a single case, this is the first report of eCR to zolbetuximab and chemotherapy in a patient with CLDN18.2‐positive GC. This case report suggests that chemotherapy combined with zolbetuximab may offer greater therapeutic benefit than chemotherapy combined with immune checkpoint inhibitors in patients with undifferentiated GC. Further analysis is expected to enable the identification of patients with CDLN18.2‐positive GC in whom zolbetuximab treatment is effective.

## Conflicts of Interest

The authors declare no conflicts of interest.

## Consent

Consent for publication of the case was provided by the patient's family.

## Supporting information




**FIGURE S1**: Upper endoscopy reveals overall shrinkage of the primary lesion, with some areas accompanying ulceration (arrowheads).

## Data Availability

The authors have nothing to report.
