# Supplementary figures and images for: Endoscopic Complete Response to Zolbetuximab for Advanced Gastric Cancer With Claudin 18.2 Positive: A Case Report
Source: DEN Open. 2025 Aug 22;6(1):e70189. doi: 10.1002/deo2.70189 (PMC12371464; doi:10.1002/deo2.70189)

## Slide 1
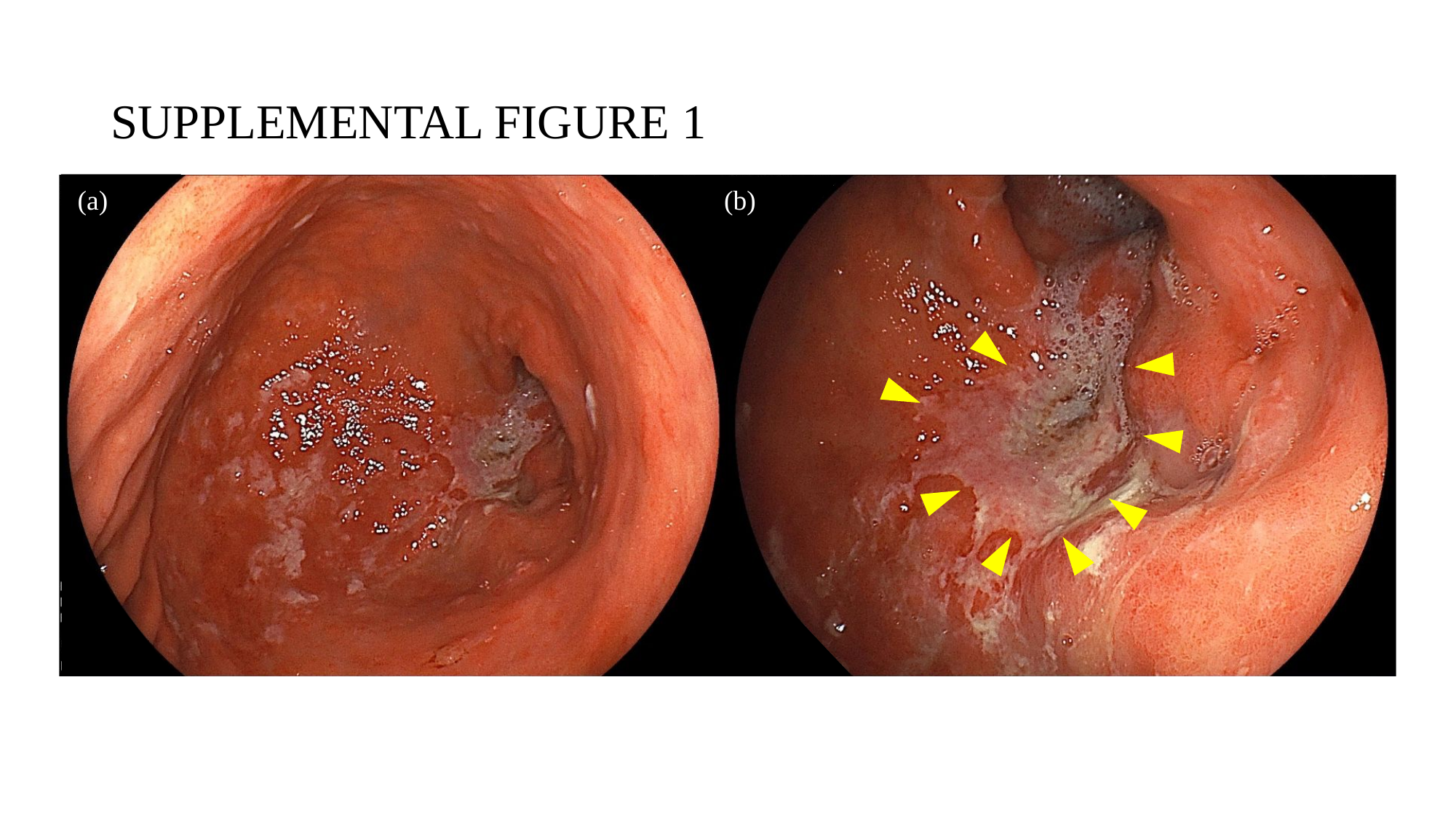

# SUPPLEMENTAL FIGURE 1
(a)
(b)

Supplement: Supplementary file 1 — FIGURE S1: Upper endoscopy reveals overall shrinkage of the primary lesion, with some areas accompanying ulceration (arrowheads). [file DEO2-6-e70189-s001.pptx]
